# Supplementary material for: Live-cell single particle imaging reveals the role of RNA polymerase II in histone H2A.Z eviction
Source: eLife. 2020 Apr 27;9:e55667. doi: 10.7554/eLife.55667 (PMC7259955; doi:10.7554/eLife.55667)
Supplement: Supplementary file 1. [file elife-55667-supp1.docx]

**Supplementary file 1:** List of strains used in this study

| STRAIN | GENOTYPE | Fig. | SOURCE |
| --- | --- | --- | --- |
| W1588-4C | *MATa ade2-1 can1-100 his3-11,15 leu2-3,112 trp1-1 ura3-1 RAD5+* |  | Tsukiyama lab |
| H2A.Z-Halo | *W1588-4C ADE+ HTZ1-HALO-KanMx pdr5::hphMX6* | 1 | This work |
| Swr1-Halo | *W1588-4C ADE+ SWR1-HALO-KanMx pdr5::hphMX6* | 1 | This work |
| Swr1-Halo, swc2∆ | *W1588-4C ADE+ SWR1-HALO-KanMx pdr5::hphMX6 swc2::ClonNat* | 1 | This work |
| FY406 | *MATa (hta1-htb1)∆::LEU2, (hta2 htb2)∆::TRP1, his3∆200 leu2∆1 ura3-52 trp1∆63 lys2-128∆ <pSAB6 (HTA1-HTB1-URA3)* |  | Winston lab |
| Halo-H2B | *MATa (hta1-htb1)∆::LEU2, (hta2-htb2)∆::TRP1, his3∆200 leu2∆1 ura3-52 trp1∆63 lys2-128∆ <pJH55 (HTA1-Halo-HTB1-HIS3)* | 1 | This work |
| Free Halo | *MATa tor1-1 fpr1::loxP-LEU2-loxP RPL13A-*  *2****x****FKBP12::loxP pdr5::loxP Swc5-FRB-GFP KanMx*  *<pRS416-NLS-Halo* | S1.1 | This work |
| H2A.Z-Halo, Swc5-FRB | MATa tor1-1 fpr1:LEU2mRPL13A-2**x**FKBP12 pdr5::loxP HTZ1-Halo-NatMX SWC5-FRB-hphMX6 | 2 | This work |
| H2A.Z-Halo, Swc5-FRB, Rpb1-FRB | MATa tor1-1 fpr1:LEU2mRPL13A-2**x**FKBP12 pdr5::loxP HTZ1-Halo-NatMX SWC5-FRB-hphMX6 RPB1-FRB-GFP-KanMx | 3 | This work |
| H2A.Z-Halo, Swc5-FRB, Ino80-FRB | MATa tor1-1 fpr1:LEU2mRPL13A-2**x**FKBP12 pdr5::loxP HTZ1-Halo-NatMX SWC5-FRB-hphMx6 INO80-FRB-GFP-KanMx | 3 | This work |
| H2A.Z-Halo, Swc5-FRB-GFP | MATa tor1-1 fpr1:LEU2mRPL13A-2**x**FKBP12 pdr5::loxP HTZ1-Halo-NatMX SWC5-FRB-GFP-KanMx | S3.1 | This work |
| H2A.Z-Halo, Swc5-FRB-GFP,  Rpb1-FRB | MATa tor1-1 fpr1:LEU2mRPL13A-2**x**FKBP12 pdr5::loxP HTZ1-Halo-NatMX SWC5-FRB-GFP-KanMx, RPB1-FRB-hphMX6 | S3.1 | This work |
| H2A.Z-Halo, Rpb1-FRB | MATa tor1-1 fpr1:LEU2mRPL13A-2**x**FKBP12 pdr5::loxP HTZ1-Halo-NatMX RPB1-FRB-GFP-KanMx | S3.2 | This work |
| H2A.Z-Halo, Ino80-FRB | MATa tor1-1 fpr1:LEU2mRPL13A-2**x**FKBP12 pdr5::loxP HTZ1-Halo-NatMX INO80-FRB-GFP-KanMx | S3.2 | This work |
| H2A.Z-Halo, Ctk1-FRB, Swc5-FRB | MATa tor1-1 fpr1:LEU2mRPL13A-2**x**FKBP12 pdr5::loxP HTZ1-Halo-NatMX SWC5-FRB-hphMX6 CTK1-FRB-GFP-KanMx | 4 | This work |
| H2A.Z-Halo, Kin28-FRB, Swc5-FRB | MATa tor1-1 fpr1:LEU2mRPL13A-2**x**FKBP12 pdr5::loxP HTZ1-Halo-NatMX SWC5-FRB-hphMX6 KIN28-FRB-GFP-KanMx | 4 | This work |
| H2A.Z-Halo, BUR1-FRB, Swc5-FRB | MATa tor1-1 fpr1:LEU2mRPL13A-2**x**FKBP12 pdr5::loxP HTZ1-Halo-NatMX SWC5-FRB-hphMX6 BUR1-FRB-GFP-KanMx | 4 | This work |
| H2A.Z-Halo, CET1-FRB, Swc5-FRB | MATa tor1-1 fpr1:LEU2mRPL13A-2**x**FKBP12 pdr5::loxP HTZ1-Halo-NatMX SWC5-FRB-hphMX6 CET1-FRB-GFP-KanMx | 4 | This work |
