# Supplementary material for: Live-cell single particle imaging reveals the role of RNA polymerase II in histone H2A.Z eviction
Source: eLife. 2020 Apr 27;9:e55667. doi: 10.7554/eLife.55667 (PMC7259955; doi:10.7554/eLife.55667)
Supplement: Supplementary file 2. [file elife-55667-supp2.docx]

**Supplementary file 2:** List of results from MSD and Spot-On analysis

Results from Fig. 2

|  | MSD | | | Spot-On | | |
| --- | --- | --- | --- | --- | --- | --- |
|  | D bound  (µm^2^ s^-1^ ) | D free  (µm^2^ s^-1^ ) | % bound | D bound  (µm^2^ s^-1^ ) | D free  (µm^2^ s^-1^ ) | % bound |
| H2A.Z  SWC5-FRB  [-RAP] | 0.087 ± 0.003 | 1.403 ± 0.160 | 77.9 ± 1.96 | 0.032 ± 0.0002 | 1.339 ± 0.010 | 79.1 ± 0.07 |
| H2A.Z  SWC5-FRB  [+RAP] | 0.087 ± 0.005 | 1.149 ± 0.085 | 44.0 ± 2.63 | 0.047 ± 0.0005 | 1.850 ± 0.009 | 49.2 ± 0.00 |

Results from Fig. 3

|  | MSD | | | Spot-On | | |
| --- | --- | --- | --- | --- | --- | --- |
|  | D bound  (µm^2^ s^-1^ ) | D free  (µm^2^ s^-1^ ) | % bound | D bound  (µm^2^ s^-1^ ) | D free  (µm^2^ s^-1^ ) | % bound |
| H2A.Z  RPB1-FRB  SWC5-FRB  [+RAP] | 0.090 ± 0.004 | 1.027 ± 0.092 | 77.9 ± 1.96 | 0.040 ± 0.0003 | 1.533 ± 0.008 | 65.6 ± 0.07 |
| H2A.Z  INO80-FRB  SWC5-FRB  [+RAP] | 0.112 ± 0.008 | 1.238 ± 0.089 | 46.0 ± 2.90 | 0.056 ± 0.0007 | 1.939 ± 0.009 | 47.4 ± 0.09 |

Results from Fig. 4

|  | MSD | | | Spot-On | | |
| --- | --- | --- | --- | --- | --- | --- |
|  | D bound  (µm^2^ s^-1^ ) | D free  (µm^2^ s^-1^ ) | % bound | D bound  (µm^2^ s^-1^ ) | D free  (µm^2^ s^-1^ ) | % bound |
| H2A.Z  KIN28-FRB  SWC5-FRB  [+RAP] | 0.081 ± 0.004 | 1.383 ± 0.123 | 64.0 ± 2.57 | 0.033 ± 0.0003 | 1.599 ± 0.007 | 64.7 ± 0.07 |
| H2A.Z  BUR1-FRB  SWC5-FRB  [+RAP] | 0.102 ± 0.006 | 1.172 ± 0.228 | 46.8± 7.56 | 0.052 ± 0.0006 | 1.906 ± 0.008 | 49.4 ± 0.07 |
| H2A.Z  CTK1-FRB  SWC5-FRB  [+RAP] | 0.087 ± 0.005 | 1.126 ± 0.111 | 47.1 ± 4.20 | 0.047 ± 0.0005 | 1.669± 0.007 | 47.2 ± 0.08 |
| H2A.Z  CET1-FRB  SWC5-FRB  [+RAP] | 0.112 ± 0.009 | 1.374 ± 0.113 | 45.6 ± 3.11 | 0.051 ± 0.0006 | 1.834 ± 0.007 | 48.0 ± 0.08 |
